# Practical Notes on Diagnosis and Treatment

**Published:** 1908-07-11

**Authors:** 


### Practical Notes on Diagnosis and Treatment.

**Insomnia.**

In practice you may be sure you will find free and long daily exposure to sunshine a valuable adjuvant in the cure of insomnia.—*Sir James Sawyer.*

**Vomiting of Pregnancy.**

One minim of tincture of iodine in an ounce of water to be repeated every two hours until vomiting ceases is often promptly successful.—*Dr. A. H. W. Ayling.*

**Neuralgia.**

Ten or twelve minims of tincture of gelsemium with or without a few grains of bromide in a mixture is an excellent temporary remedy for neuralgia. The patient should be warned that the relief obtained does not warrant him in neglecting any source of obvious irritation, such as a carious tooth.

**Hæmaturia in Children.**

There is little doubt that hæmaturia in childhood occurs apart from nephritis and that under appropriate treatment the renal disturbance, whatever it be, readily subsides. Leeches or cupping are useful, and rest, milk diet, and calcium chloride should be prescribed.

**Tubercle of the Tongue.**

A tubercular ulcer of the tongue is usually shallow, superficial, with well-defined edges and a "granulated" base. It is situated on the dorsum or border of the tongue, and does not cause much pain except in the act of mastication.—*Dr. Andrew Wylie.*

**Sciatica.**

It is in sciatica, of all the neuralgias, that massage has won its greatest reputation. Truly astonishing results have been obtained, even when the affection has been of many years' standing, and after every other conceivable means of relief has proved unsuccessful.—*Dr. Lee.*

**Albuminuria and Exercise.**

In a series of examinations made on members of the College boat clubs at Oxford, Dr. Collier found that the urine collected from one to two hours after rowing frequently contained albumen. In some instances this was true of every member of the crew. The more energetic the exercise the more marked were these results.

**Sore Throat in Secondary Syphilis.**

In the very early stages of this condition one or more clear vesicles are sometimes seen on the fauces and soft palate; these may persist as such for some time before breaking down into the familiar "snail track" ulcer. In a typical case they are about as large as a chicken-pox vesicle.

**Lunacy Certificates.**

After efficiency, brevity is one of the most valued attributes of a lunacy certificate. Nevertheless, desirable as it is, brevity should not be attained at the expense of adequacy, nor should it be sought for outside the rules of ordinary syntax.—*Dr. Leonard Williams.*

**Staining for Tubercle Bacilli.**

Not staining long enough is a frequent cause of failures. Five minutes is safest. Remember that, practically speaking, the object of decolourising is to make the background a different colour to the bacillus. When the former shows rose-pink (after washing) enough has been done.

**Sub-acute and Chronic Gout.**

Guaiacum resin is extremely valuable in treating the chronic form of gout, and in preventing their recurrence. Five grains of the resin (in a cachet) should be given thrice daily and the dose increased by one grain per dose each week until a maximum of 10 grains is reached; this should be continued for six months.—*Dr. A. P. Luff.*

**Glaucoma.**

Much can be done both for the pain and for the pathological condition in cases of glaucoma by very gentle massage of the eyeball through the upper lid. In acute cases the patient may thus be treated in the interval before an operation can be undertaken; in chronic it sometimes suffices to repeat the process every few days and to order eserine drops.

**Flavouring Agents.**

Speaking broadly, children and women prefer sweet flavours, men (especially smokers) bitter and warm aromatic flavours. Most children also like acid preparations, but abhor bitter things. It is questionable, indeed, if bitters, as such, are of the slightest value in the treatment of children.—*Prof. C. R. Marshall.*

